# Sexual dimorphism in metabolomic and phenotypic spectra of UGT deficiency: findings from the Canadian Longitudinal Study on Aging

**DOI:** 10.1186/s13293-025-00708-5

**Published:** 2025-04-22

**Authors:** Ana Lucia Rivera-Herrera, Michèle Rouleau, Mahukpe Narcisse Ulrich Singbo, Tania Cuppens, Julien Prunier, Arnaud Droit, David Simonyan, Chantal Guillemette

**Affiliations:** 1 Faculty of Pharmacy, Université Laval, Québec, QC G1V 0A6 Canada; 2 Université Laval Cancer Research Center, Québec, QC G1R 3S3 Canada; 3 Centre Hospitalier Universitaire (CHU) de Québec Research Center - Université Laval, R4701.5, 2705 Blvd Laurier Québec, Québec, QC G1V 4G2 Canada; 4 Faculty of Medicine, Department of Molecular Medicine, Université Laval, Québec, QC G1V 0A6 Canada

**Keywords:** CLSA, Metabolomics, Glycosyltransferase, Human gene knockout, Metabolic disorders

## Abstract

**Background:**

Two of the most frequently deleted genes in the human genome are the UDP-glycosyltransferases *UGT2B17* and *UGT2B28.* They encode metabolic enzymes of the glucuronidation pathway that plays a pivotal role in the maintenance of cellular homeostasis for a variety of small molecule metabolites. These deletions may impact health, yet their effects remain poorly understood. We evaluated the impact of UGT deficiency on the plasma metabolome and examined the association between altered metabolites and health outcomes.

**Methods:**

The metabolomic profiles of 4262 proficient gene carriers were compared with those of 352 *UGT2B17*-deficient, 97 *UGT2B28*-deficient, and 20 double-gene-deficient individuals from the Canadian Longitudinal Study on Aging. Significant metabolites found in these comparisons were analyzed for their associations with common diseases.

**Results:**

The unexpectedly broad molecular divergence found in *UGT*-deficient metabolomes, which affected > 10% of metabolites, implies their significant influence across various metabolite classes—particularly lipids and amino acids — extending beyond their known substrates. The metabolic profiles of *UGT2B17*-deficient men and *UGT2B28*-deficient women were most impacted, with *UGT2B17* deficiency affecting various metabolites linked to metabolic diseases, arthritis, and osteoporosis. Metabolites impacted by a *UGT2B28* deficiency such as amino acids, were linked to metabolic disorders in women.

**Conclusion:**

The findings significantly advance our understanding of the metabolic landscape associated with these frequently deleted genes in the human genome, which may influence susceptibility to various diseases in a sex-specific manner, laying the groundwork for determining their pathological mechanisms and impact on human health.

**Supplementary information:**

The online version contains supplementary material available at 10.1186/s13293-025-00708-5.

## Highlights

Divergent metabolomes of deficient glycosyltransferase individuals (UGT KO).

Highest impact of UGT deficiency on lipid profiles.

Unique metabolic signatures characterize *UGT2B17* KO men and *UGT2B28* KO women.

UGT KO metabolic signatures are linked to a variety of diseases.

## Plain language summary

The human body constantly produces a variety of small molecules, including steroids, bile acids, fatty acids, and hormones. UDP-glycosyltransferases (UGTs) are a key family of enzymes that help balance these molecules and remove them from the body through a process called glucuronidation. Interestingly, two UGT genes, UGT2B17 and UGT2B28, are often naturally deleted, meaning some people are born without these genes. This gene absence results in a complete deficiency of the associated UGT proteins, potentially disrupting the balance of certain metabolites in the body. To understand how missing UGT2B17 or UGT2B28 affects the body’s full profile of metabolites (known as the circulating metabolome), we compared individuals without one or both of these genes (deletants) to those with both gene copies (references). We found distinct, sex-specific changes in metabolite levels: men lacking UGT2B17 had higher levels of steroids and phospholipids, while women lacking UGT2B28 had lower levels of fatty acids. Additionally, these disrupted metabolite levels were linked to different diseases in a sex-specific way. These insights help clarify how these commonly deleted genes influence metabolism and may lead to disease, opening avenues for future health research.

## Background

The blood metabolome mirrors the biochemical activities of different organs and exhibits significant variation between sexes [1]. When combined with genomics, metabolomics has the potential to uncover gene functions and their influence on different aspects of health and disease [2]. Although single nucleotide polymorphisms account for the majority of base-pair variations in human genomes, a small subset of genes undergoes complete germline deletion (herein referred to as knockout, KO), with largely unclear metabolomic and functional implications. Individuals with natural gene KOs offer a unique opportunity to investigate overall gene functions, metabolomic characteristics, and their associations with human health and disease [3–5].

Relevant to metabolomic changes, metabolic genes have been identified to harbor adaptive structural variants including complete gene deletions, the frequency of which varies among different ethnic populations [3–5], possibly because of differences in diet [6]. The UDP-glycosyltransferase genes *UGT2B17* and *UGT2B28* stand out because they frequently undergo germline whole-gene deletion, leading to a complete deficiency [3, 5]. In fact, they represent two of the most frequently deleted genes in the human genome [5]. The frequency of *UGT2B17* KO varies greatly from 9% for Caucasians to > 70% for Asians [3, 5]; for *UGT2B28* KO, the frequency ranges from 2 to 3% for Caucasians and Asians to 10% for Africans [4, 7]. Individuals with either germline KO do not express the corresponding UGT2B17 or UGT2B28 proteins in any tissue, including metabolic-intensive organs such as the liver [8–10]. Conversely, gene-proficient individuals express UGT2B17 and UGT2B28 in various tissues, including the metabolically active liver, kidneys, gastrointestinal tissues, bladder and a variety of other organs and cells [11].

UGTs encode enzymes of the glucuronidation pathway that utilize sugar nucleotides as co-substrates. This pathway plays a pivotal role in preserving cellular homeostasis and protecting against drugs and other foreign substances. It regulates the cellular abundance (and thus bioactivity) of endogenous metabolites such as bilirubin and sex steroids [12–16], which is fundamental to cellular metabolic pathways. Moreover, UGTs participate in pharmacokinetics, contributing to the inactivation of more than 50% of commonly prescribed medications and consequently influencing target-site exposure pharmacological effects [12, 17]. A deficiency in a UGT enzyme, such as the bilirubin-conjugating UGT1A1, can be detrimental in humans, leading to the accumulation of unconjugated neurotoxic bilirubin, which is characterized by liver dysfunction [18]. For the UGT2B17 and UGT2B28 enzymes, the absence of their genes is not lethal; however, an increasing number of studies have found that differences in their expression patterns and/or deletions are associated with osteoporosis, autoimmune diseases and cancers [19–29]. However, the underlying mechanisms and the potentially affected metabolites in these conditions remain unknown. There are few known metabolite substrates for these enzymes, including androgenic steroids, with UGT2B28 being particularly understudied [30–33]. Thus, the detailed spectrum of metabolites that serve as substrates or influenced by the UGT2B17 and UGT2B28 metabolic pathways remains unknown.

Our goal was to enhance our understanding of the metabolic processes associated with the UGT2B17 and UGT2B28 pathways. We analyzed population-based genomic and metabolomic data from the Canadian Longitudinal Study on Aging (CLSA) [34]. This large cohort includes numerous *UGT* KO individuals, enabling an examination of genetically influenced systemic metabolomic profiles and evaluation of whether the metabolites affected by these genes are linked to common age-related conditions. We hypothesized that the absence of the UGT2B17/UGT2B28 metabolic pathway could lead to alterations in metabolite levels, resulting in significant systemic changes. We suggest that these metabolic changes may affect disease susceptibility. Findings provide evidence for divergent influences of *UGT* deletions on the plasma metabolome of males and females, with unexpected metabolic changes, which were particularly notable for *UGT2B17* KO males and *UGT2B28* KO females. The impacted metabolites were linked to prevalent health conditions and chronic diseases, namely obesity, diabetes, hypertension, osteoporosis and arthritis.

## Methods

### Sex as a biological variable

The study included an equal number of male and female participants. Both overall and sex-based analyses were conducted, and we report the sex-related influence of *UGT* KOs on the circulating metabolome.

### Study population

The CLSA is a longitudinal study established to study the genetic and environmental contributions to human health and diseases and determinants of aging by collecting information on the changing biological, medical, psychological, social, lifestyle and economic aspects of participants’ lives. It comprises 51,338 community-dwelling Canadians aged 45–85 years at recruitment that are prospectively followed every 3 years for at least 20 years or until death [34]. Detailed descriptions of the study population, protocols and data collection have been reported and may be accessed online [35]. The current study focused on baseline genetics and metabolomic data derived from blood samples of the comprehensive CLSA cohort (30,097 participants) collected between 2010 and 2015. The CLSA participants were originally randomly selected from within 25–50 km of 11 data-collection sites in seven Canadian provinces.

### Genotyping

Blood samples were collected as previously described [36]. Whole blood buffy coats were isolated from peripheral blood drawn into EDTA vacutainers, following centrifugation at 2000 *× g* for 10 minutes and removal of the plasma layer. Samples were immediately moved to -80°C storage. The time from blood collection to -80°C storage was under two hours for all participants. Genomic DNA was extracted from buffy coat samples using the purification protocol ”Chemagic DNA Buffy Coat Kit special 200µl prefilling VD151007” on the Chemagic MSM I instrument (Perkin-Elmer article No. CMG-533). The detailed procedure may be accessed online [37]. A total of 26,622 individuals from the CLSA comprehensive cohort were genotyped across 794,409 genetic markers using the Affymetrix UK Biobank Axiom as well as 308 million genetic variants imputed from the Trans-Omics for Precision Medicine program (TOPMed) reference panel as previously described [36]. Data were available in the binary PLINK format (Affymetrix UK Biobank Axiom Array) and BGEN V.1.2 format (TOPMed). PLINK software was used for SNP extraction and assessment of allele frequencies. The occurrence of *UGT2B17* and *UGT2B28* deletions was established based on the tag SNPs rs2708666 and rs11249532, which are in linkage disequilibrium with the respective deletions [5] and imputed from the genomic data. The *UGT2B17* KO group comprised participants with the rs2708666A/A homozygous genotype who were homozygous carriers of *UGT2B28* (tagged by rs11249532A/A). The *UGT2B28* KO group comprised those with the rs11249532T/T homozygous genotype (e.g., absence of both *UGT2B28* copies) who were homozygous carriers of *UGT2B17* (rs2708666G/G). Participants with both rs2708666A/A and rs11249532T/T comprised the double KO group. The reference group comprised participants with homozygous rs2708666G/G and rs11249532A/A genotypes, thus carrying both copies of *UGT2B17* and *UGT2B28*.

### Metabolomics

Metabolomics analyses were conducted using plasma collected by centrifugation of whole blood collected in EDTA vacutainers and stored at − 80 °C within 2 h of blood collection as described above. Untargeted global metabolomic profiling was conducted for 9992 CLSA participants selected from the Comprehensive cohort to be representative of each data collection site, age groups and sex. Briefly, 1458 metabolites were quantified using the ultra-HPLC–tandem mass spectroscopy HD4 platform of Metabolon Inc. Detailed descriptions for sample preparation, metabolomics data acquisition and processing may be accessed online [38]. Metabolite levels of each sample were normalized using a pool of well-characterized EDTA plasma quality control (QC) samples used throughout the analysis of the dataset (QC-normalization). Missing values were imputed with the minimum value. Classification of metabolites within superpathways, subpathways and subclasses were as assigned by Metabolon Inc. Xenobiotics (*n* = 218) and metabolites measured in fewer than 50% of individuals (*n* = 147) were excluded from further analysis, leaving a total of 1093 metabolites for subsequent analysis, as detailed in Supplementary Fig. 1.

### Lifestyle, physical measures and chronic diseases

Baseline sociodemographic data of CLSA participants included in this study were the age at enrolment, sex, smoking and alcohol intake. Smoking status was classified as current (daily smoker), occasional (former daily smoker/never a daily smoker or has smoked less than 100 cigarettes in a lifetime), former (former daily and former occasional smoker) and never smoker. Alcohol intake was classified as current (almost every day, 4–5 times per week, 2–3 times per week, once per week, 2–3 times per month and about once a month), occasional (less than once a month) and non-drinkers (never).

Baseline physical measures were collected by trained CLSA staff following standard operating procedures. BMI (kg/m^2^) was calculated in terms of body weight (kg) and height (m). The list of diseases was taken from the self-reported physician-diagnosed chronic diseases included in the CLSA questionnaires that existed at the time of reporting. Some of the reported diseases were combined following recommendations [39] and ICD-10 codes, which groups diseases according to sufficient pathophysiological similarity. Disease categories are described in Supplementary Table 1.

### Statistics

Levels of measured metabolites were natural log-transformed prior to statistical analysis. Fold-change values were determined based on the mean of each group compared to the reference group. An additional analysis was performed exclusively on postmenopausal women, as the number of premenopausal women in the study was too limited. Relevant details are provided in the figure legends where applicable. A one-way analysis of variance *F*-test was used to compare the means of the different groups. Statistical significance was determined with unadjusted and Tukey-adjusted *P*-values, and both are reported in Supplementary Tables 2 and 2.1. Pathway enrichment analysis of affected metabolites was carried out according to the classification of 785 metabolites (Supplementary Fig. 1). Enrichment scores were calculated as follows [7]:

Enrichment Score = (k/m)/((n-k)/(N-m)).

m = number of metabolites in the pathway.

k = number of significant metabolites in the pathway.

n = total number of significant metabolites.

N = total number of metabolites.

For categorical phenotype metabolic data, the Chi-square test was performed, with *P*-values either not adjusted or adjusted for false-discovery rate. For continuous phenotype data, the one-way analysis of variance *F*-test with unadjusted and Tukey-adjusted *P*-values was performed. Univariate and multivariate logistic regression analyses were conducted to estimate odds ratios and 95% confidence intervals for the association between chronic conditions as dependent categorical variables and significantly altered metabolites as independent variables. Covariables used in the multivariate model included age, smoking status, alcohol consumption and sex in the overall analyses. Additionally, a secondary model included menopausal status as well as ever-use of hormone replacement therapy (HRT) as covariables. Details are provided in the figure legends. A two-way ANOVA was used to examine the effect of UGT KOs and sex on metabolite levels. The associations between significantly changed metabolites (as independent variables) and blood biomarkers (as dependent variables) were evaluated using linear regressions. Multiple linear regressions, incorporating the same covariates as in the logistic regression analyses (with categorical variables encoded using one-hot encoding), were then used to adjust these associations. The lm function in R was used for these analyses. Analyses were conducted using R (version 4.4.0).

## Results

### Study cohort

**Fig. 1:**
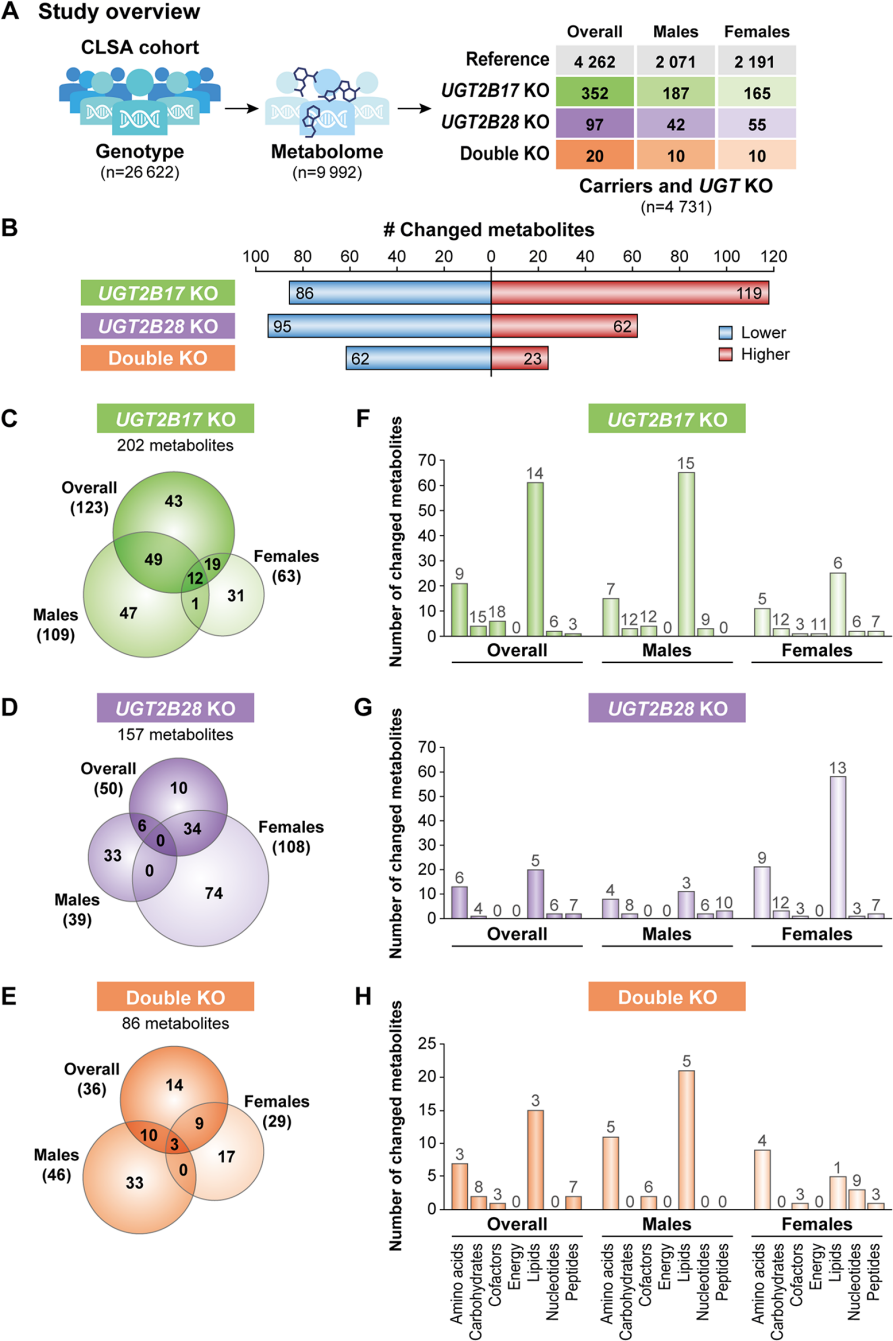
UGT deletions significantly impact the metabolic profile of plasma. (**A**) The study cohort consisted of homozygous reference gene carriers or *UGT* knockout (KO) individuals (*n* = 4731) from the genotyped comprehensive CLSA cohort for which metabolomics profiles (*n* = 1458 metabolites) were assessed. (**B**) Number of significantly altered metabolites in each *UGT* KO group relative to the gene-proficient reference group (*P* < 0.05). Total number of metabolites of lower or higher abundance is given for each *UGT* KO group. A detailed report on the metabolites affected in each group is available in Supplementary Table 2. **C–E**. Venn diagrams depicting the number of altered metabolites in the overall and sex-based analyses; (**C**) *UGT2B17* KO, (**D**) *UGT2B28* KO and (**E**) double KO. **F–H.** Distribution of altered metabolites within superpathways for each *UGT* KO group in the overall and sex-based analyses. Results were consistent when limited to postmenopausal women (Supplementary Table 2.1). The numbers above each bar represent the percentage of altered metabolites relative to the total number of metabolites measured in each superpathway

Among the 9992 CLSA participants with available metabolomics data (Fig. 1A), 469 individuals had homozygous deletion (i.e., KO) of *UGT2B17* (*n* = 352) or *UGT2B28* (*n* = 97) or both genes (*n* = 20); these individuals were compared to a reference group of 4262 subjects who were homozygous for both genes (Fig. 1A). The prevalence of these deletions in Caucasians (∼9% for *UGT2B17*, ∼3% for *UGT2B28*) aligns with previous reports [3–5]. The frequency of simultaneous deletion of both genes was ∼0.2%. Table 1 lts the main characteristics of the study groups, with nearly equal numbers of men and women with similar body mass index (BMI) ranging from 26.9 to 28.2 kg/m² and an average age of 62.8 years at the time of blood collection. Most participants were Caucasian (98%). As anticipated given the allele frequencies [3], participants of Asian and other ethnic backgrounds were represented in the KO groups. Additional details are provided in Supplementary Table 3, including the prevalence of various diseases among reference and KO groups.

**Table 1:** Characteristics of the study cohort

	Reference	*UGT2B17* KO	*UGT2B28* KO	Double KO
Overall (*n* = 4731)	4262	352	97	20
Age	62.8 ± 10.0	62.4 ± 10.0	63.9 ± 10.6	60.9 ± 10.5
BMI	28.1 ± 5.4	27.9 ± 5.7	28.2 ± 5.2	26.9 ± 5.7
Males (*n* = 2310)	2071	187	42	10
Age	62.9 ± 10.0	62.7 ± 10.0	65.5 ± 10.5	57.8 ± 9.2
BMI	28.3 ± 4.8	28.0 ± 5.4	27.4 ± 3.8	26.3 ± 1.8
Females (*n* = 2421)	2191	165	55	10
Age	62.7 ± 10.0	62.0 ± 10.1	62.7 ± 10.7	64.0 ± 11.4
BMI	27.9 ± 6.0	27.8 ± 6.2	28.9 ± 6.0	27.5 ± 8.0

Mean and standard deviations (SD) are provided. Refer to Supplementary Table 3 for further details. One-way analysis of variance *F*-test and *P*-values either not adjusted or adjusted for false-discovery rate were conducted, as described in Methods. BMI, Body mass index calculated as kg/m^2^ (kg, body weight; m, body height in meters squared).

### Divergent metabolomes of subjects with UGT KOs and disparities between sexes

Of the 1093 metabolites that were consistently measured among individuals within each experimental group, 202 (18%) differed significantly for subjects with *UGT2B17* KO, 157 (14%) for *UGT2B28* KO, and 86 (8%) for the double KO group relative to the reference group (Fig. 1B–E). In the *UGT2B17* KO group, the abundance of most changed metabolites was increased compared to the reference group, whereas in *UGT2B28* KO and double KO groups, abundance decreased for most of the affected metabolites (Fig. 1B). In each KO group, only small numbers of metabolites (< 7%) were commonly affected in both males and females, indicating sexual dimorphism with respect to *UGT* KOs (Fig. 1C–E). In the *UGT2B17* KO group, the number of affected metabolites varied more for males than females (109 metabolites compared with 63 metabolites), whereas in the *UGT2B28* KO group, metabolite abundance was affected considerably more for females than males (108 metabolites vs. 39 metabolites) (Fig. 1C–E). A distinct example of sex-divergent effects in *UGT* KOs involved glucuronide metabolites, which are produced by UGT enzymes [40]. The abundance of glucuronide derivatives of deoxycholate, a bile acid, and of catechol, a tyrosine metabolite, changed substantially in each KO group, with a particularly pronounced reduction of deoxycholate glucuronide in *UGT2B17* KO males, and an increase of catechol glucuronide in *UGT2B28* KO females (*P*_*adj*_<0.05; Supplementary Table 4). However, no sex difference was apparent in most tissues with respect to *UGT2B17* or *UGT2B28* expression apart from *UGT2B17* in the liver (higher in males than females) and in the intestine and colon (higher in females than males) (Supplementary Fig. 2) [8, 9]. Metabolites belonging to the lipid, amino acid and carbohydrate superpathways were the most affected in each *UGT* KO group compared to the reference group (Fig. 1F–H; Supplementary Tables 2 and 4).

### Sex-divergent effect of UGT KOs on certain lipid classes

**Fig. 2:**
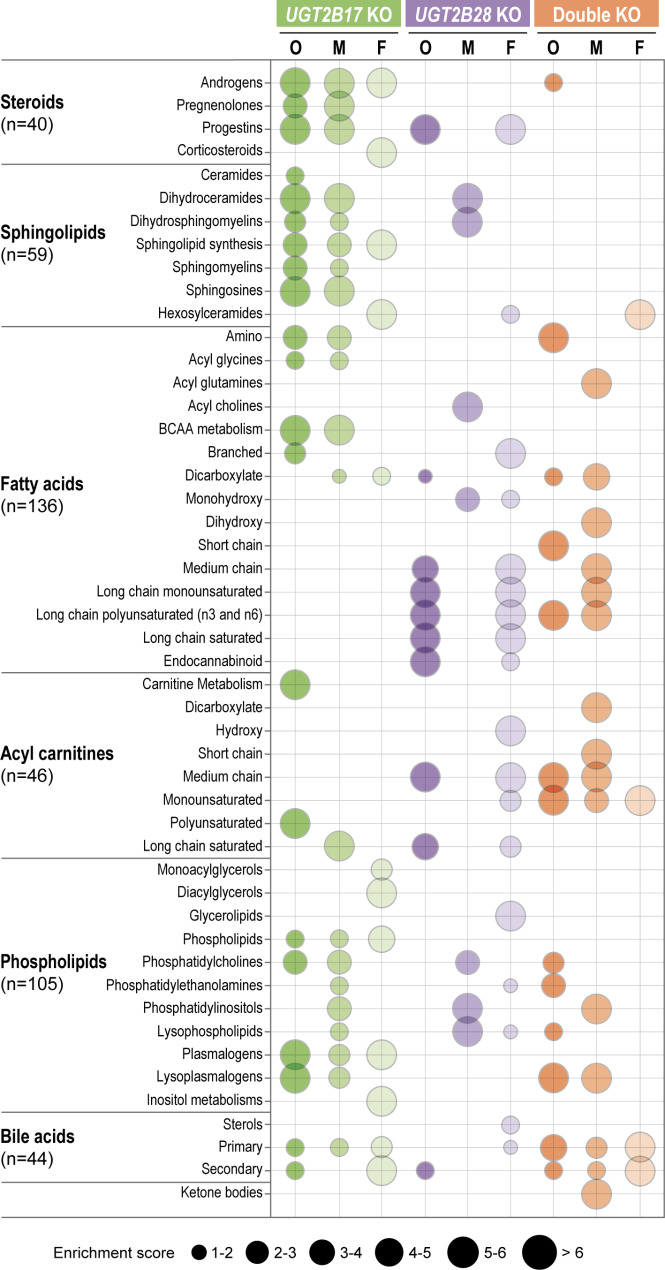
Distinct lipid subclasses are affected by each *UGT* KO. Enrichment analysis was conducted for significantly (*P* < 0.05) altered lipids in the overall (O), male (M), and female (F) analyses. Results were consistent when limited to postmenopausal women (Supplementary Table 2.1). Bubble sizes are proportional to the enrichment score. BCAA: branched-chain amino acids. Number of metabolites measured in each lipid subclass is provided

Metabolites of many lipid subclasses including cholesterol-derived steroids and bile acids, sphingolipids, fatty acids, acyl carnitines and phospholipids were higher in *UGT2B17* KO males and lower in *UGT2B28* KO females (Fig. 2, Supplementary Table 4). For *UGT2B17* KO males, the abundance of 15% of measured lipids was altered compared with 6% for *UGT2B17* KO females (Fig. 1F, Supplementary Fig. 3A). For *UGT2B28* KO subjects, males had far fewer differences in lipid abundance (3%) than females (13%) (Fig. 1G, Supplementary Fig. 3B).

**Fig. 3:**
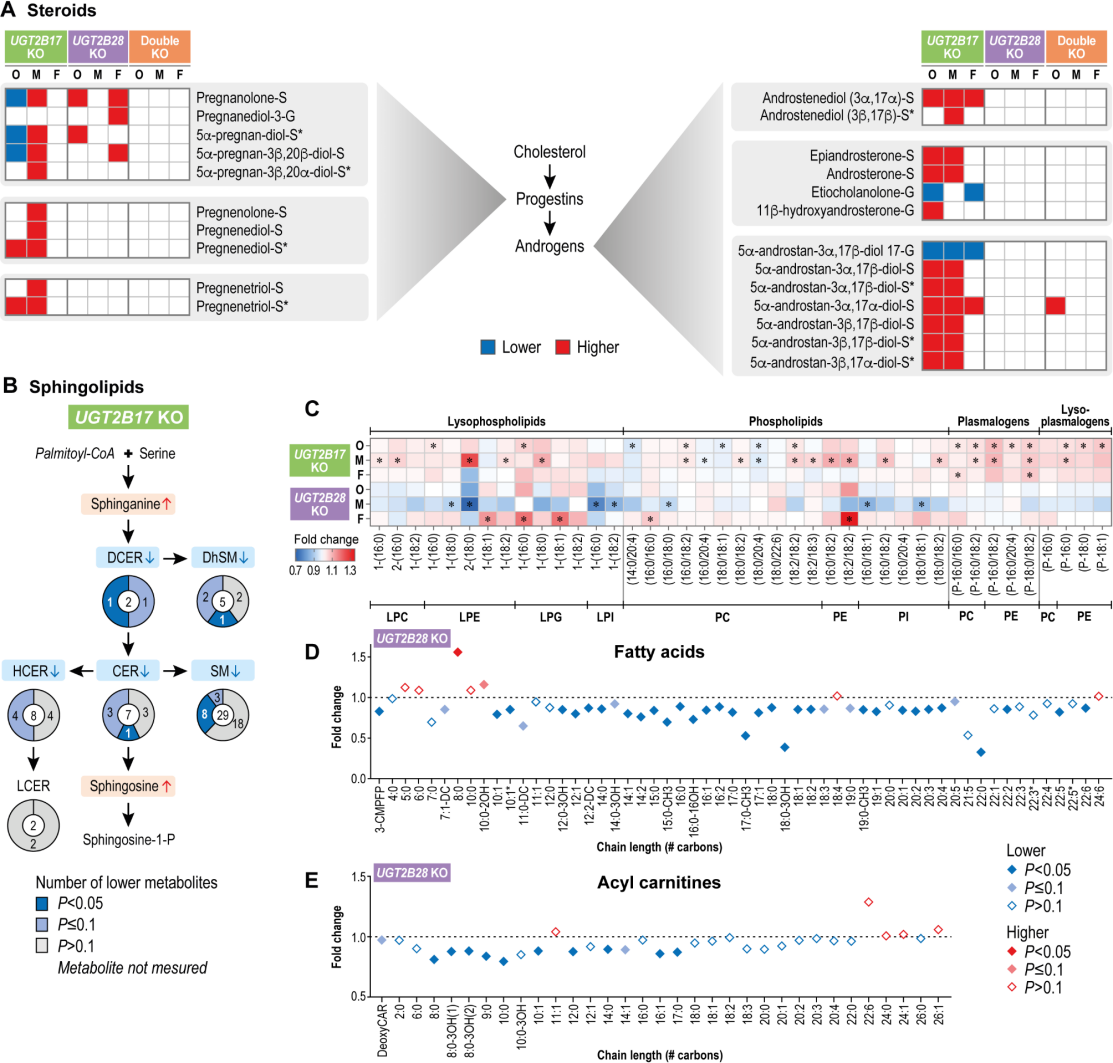
Lipid subclasses most affected by *UGT* KO. **A**. Schematic pathway of cholesterol-derived progestogens and androgens. Steroids that differ significantly in at least one *UGT* KO group are shown as tile arrays depicting sense of fold change relative to the reference group (blue, lower; red, higher). Most steroids that were measured were sulfate (-S) or glucuronide (-G) conjugates. Each asterisk denotes that the steroid has two sulfate groups. O: overall; M: males; F: females. **B**. Simplified sphingolipid metabolic pathway and sphingolipids significantly altered in *UGT2B17* KO overall analysis. The global sense of the fold change for each sphingolipid subclass is indicated by color (blue, lower; light red, higher). The numbers of measured (center) and altered sphingolipids (parts of the donut) are given for each subclass. DCER, dihydroceramides; DhSM, dihydrosphingomyelins; CER, ceramides; HCER, hexosylceramides; LCER, lactosylceramides; SM, sphingomyelins. **C**. Phospholipid profiles in *UGT2B17* KO and *UGT2B28* KO groups relative to reference. LPC, lysophosphatidylcholines; LPE, lysophosphatidylethanolamines; LPI, lysophosphatidylinositol; LPG, lysophosphatidylglycerol; PC, phosphatidylcholine; PE, phosphatidylethanolamine; PI, phosphatidylinositol. *P < 0.05. (**D**) Fatty acid and (**E**) acylcarnitine profiles for *UGT2B28* KO females relative to the reference group. Fold changes are shown relative to the reference group level set at 1.0 (dashed line). The *x* axis indicates carbon chain length and includes fatty-acid dicarboxylates (DC), monohydroxy fatty acids or hydroxy acyl carnitines (2OH, 3OH, 16OH), and branched-chain fatty acids (CH3). 3-CMPFP, 3-carboxy-4-methyl-5-pentyl-2-furanpropionate; CAR, carnitine. The terms 22:3* and 22:5* denote omega-6 polyunsaturated fatty acids

Sex steroids, and more specifically several androgens, were significantly elevated in *UGT2B17* KO males, consistent with the reported enzymatic specificity of UGT2B17 (*P*_*adj*_<0.05; Fig. 2, Supplementary Tables 2 and 4). Among the 40 sex steroids we measured, 50% were altered, with all but one more abundant, resulting in greater androgenic exposure in the *UGT2B17* KO male group (Fig. 3A, Supplementary Tables 2 and 4) [33]. Some metabolites from this lipid sub-class were significantly affected by *UGT2B17* KO status and male sex in the interaction analysis including androsterone sulfate (*P* = 0.004), 5α-androstan-3α,17β-diol monosulfate (*P* = 0.005) and pregnanolone/allopregnanolone sulfate (*P* = 0.028). By contrast, the levels of several sphingolipids, particularly sphingomyelins, were lower compared to the reference group, whereas two precursors in the ceramide metabolic pathway, namely sphinganine and sphingosine, were elevated (Fig. 3B, Supplementary Table 2); this implied that these signaling molecules had accumulated in *UGT2B17* KO males. Several phospholipids, and particularly those belonging to the glycerophospholipid and plasmalogen classes, were also elevated in *UGT2B17* KO males (Fig. 3C and Supplementary Table 2). The limited number of altered lipids in the female *UGT2B17* KO mainly featured higher levels of sulfated androgens, sulfated bile acids, and hexosylceramides but lower levels of glucuronidated androgens, non-sulfated bile acids and tetrahydrocortisol (*P*_*adj*_<0.05; Supplementary Table 2). An 84% reduction in the levels of the primary bile acid cholic acid glucuronide and of the secondary bile acid deoxycholic acid glucuronide (a known substrate of UGT2B17) constituted the most drastic changes in *UGT2B17* KO individuals, irrespective of sex (*P*_*adj*_<0.05; Supplementary Table 4, Supplementary Fig. 4A, B). In the *UGT2B28* KO group, females (but not males) exhibited significantly lower levels of several fatty acid subtypes, particularly saturated, monounsaturated, and polyunsaturated long-chain fatty acids as well as acyl carnitines (Figs. 2 and 3D-E), from which saturated and polyunsaturated were significant at adjusted *p*-values (Supplementary Table 2). These included multiple omega-3 and omega-6 fatty acids such as arachidonic acid and docosahexanoic acid (Supplementary Table 2). The abundance of bile acids was also altered only in the female *UGT2B28* KO group, with higher levels of glucuronide derivatives of deoxycholic acid and glycochenodeoxycholic acid (Supplementary Table 2). Among steroids, only progestins were changed, and more abundant, including pregnanediol-3 glucuronide (Fig. 3A). The build-up of glucuronide derivatives in UGT2B28-deficient individuals would be counterintuitive if UGT2B28 is involved in the glucuronidation of bile acids and progestins, suggesting that UGT2B28 impedes their glucuronidation by other UGTs and/or the presence of another mechanism that mediates their accumulation in plasma. Our analysis support an interaction between *UGT2B28* KO status and female sex for a subset of metabolites including those that belong to the category of fatty acids (dodecadienoate (12:2) (*P = *0.043), 3-hydroxyoleate (*P = *0.038), behenate (22:0) (*P = *0.020) and (2 or 3)-decenoate (10:1n7 or n8) (*P = *0.011)), as well as the acyl carnitine 3-hydroxydecanoylcarnitine (*P = *0.015), the bile acid lycochenodeoxycholate glucuronide (*P = *0.015) and a metabolite part of the N-acylethanolamine family (endocannabinoid stearoyl ethanolamide (*P = *0.031)).

The lipids altered in *UGT2B28* KO males were primarily of the phospholipid subclasses, with levels of the lysophospholipid and glycerophosphoinositol classes being particularly lower, unlike in *UGT2B17* KO males (Fig. 3C). N-palmitoyl-sphinganine and dihydrosphingomyelin were reduced in *UGT2B28* KO males, similar to observations in *UGT2B17* KO males, supporting a role for these two UGTs in regulating sphingolipid signaling, particularly in men (Supplementary Table 2).

The double KO group exhibited fewer significantly altered lipids, potentially reflecting the smaller sample size of this group and hence the limited ability to detect significant changes (Fig. 1H). Males were more affected than females, like the *UGT2B17* KO group, but both males and females exhibited mostly lower lipid levels, similar to observations for the *UGT2B28* KO group (Supplementary Fig. 3C). Of note, glucuronide derivatives of cholic and deoxycholic acids were less abundant, like *UGT2B17* KO, without any sex disparity (*P*_*adj*_<0.05); Supplementary Fig. 4A, B). The testosterone sulfated derivative 5α-androstan-3α,17α-diol-sulfate was significantly higher in the double KO overall group (Fig. 3A).

### Notable changes in amino acids, carbohydrates and certain uncharacterized metabolites in *UGT2B17* KO and *UGT2B28* KO individuals

**Fig. 4:**
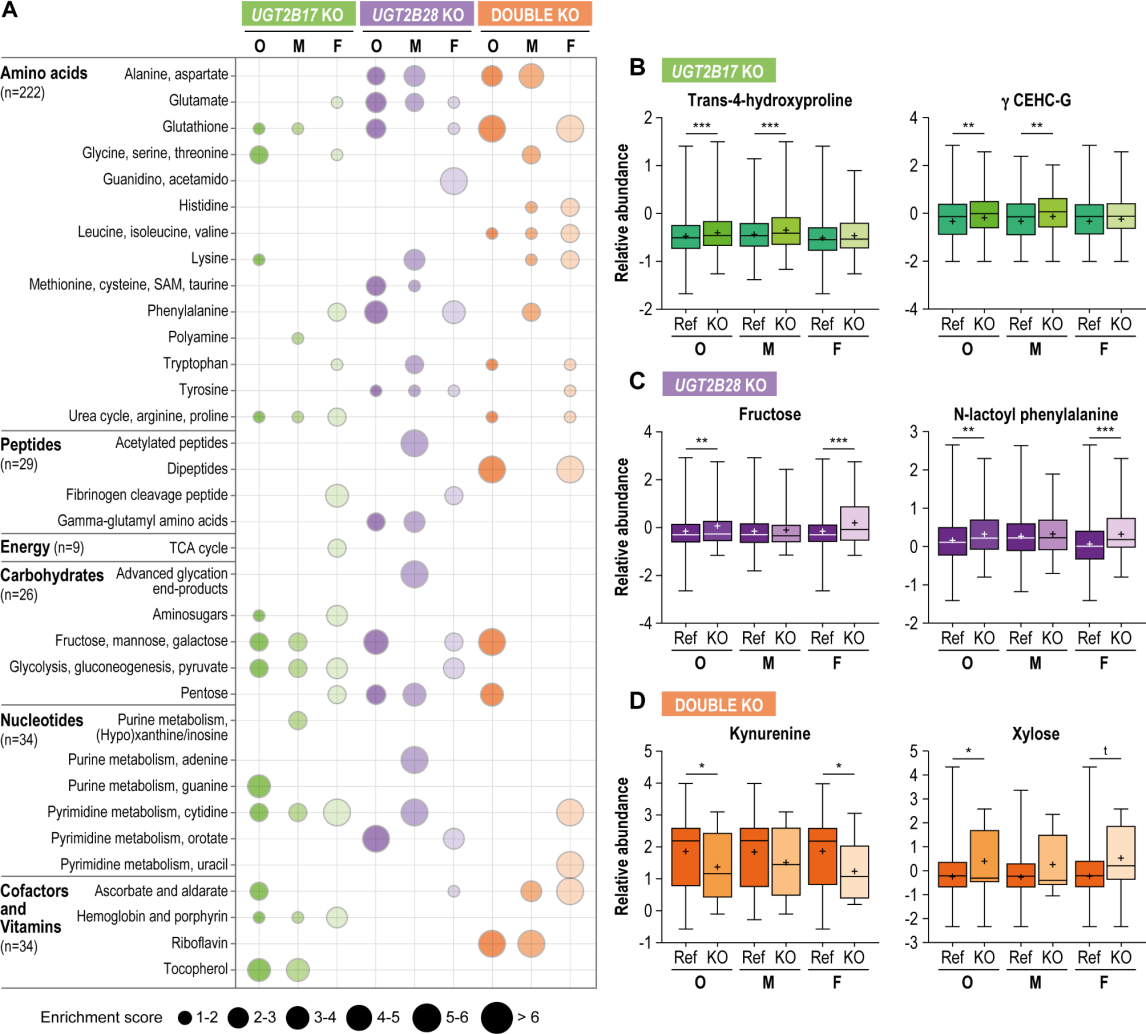
Non-lipid metabolic pathways that are altered by *UGT* KO. **A**. Enrichment analysis of each subclass of metabolites for the overall (O), male (M) and female (F) analyses. Bubble size represents the enrichment score for each *UGT* KO group. **B–D**. Relative abundance of significantly altered representative metabolites in each KO group. Results were consistent when limited to postmenopausal women (Supplementary Table 2.1). Boxes represent interquartile range, the median (horizontal bar), and mean (+). Whiskers depict minimum and maximum values. Ref: reference group. *P < 0.05, **P < 0.01, ***P < 0.001

Amino acid and carbohydrate metabolites constituted the other superpathways most affected in *UGT* KO individuals (Fig. 4A, Supplementary Table 2). The “glycine, serine and threonine”, “urea cycle and arginine and proline”, “glycolysis” and “fructose” pathways were particularly altered (Fig. 4A). Changes unique to one sex were commonly observed, with *UGT2B17* KO males characterized by higher levels of several metabolites related to the degradation of vitamin E and lower levels of bilirubin and by-products of its degradation (Fig. 4B). Furthermore, the levels of pyrimidine metabolite cytidine were significantly affected by the interaction between UGT2B17 KO status and female sex (*P* = 0.002).

*UGT2B28* KO females exhibited higher levels of fructose as well as N-lactoyl-phenylalanine, an amino acid derivative, indicative of mitochondrial dysfunction that is prevalent in plasma of individuals experiencing septic shock or with certain health conditions such as diabetes [41, 42] (*P*_*adj*_<0.05; Fig. 4C; Supplementary Tables 2, 4.1). The effect of *UGT2B28* KO and female sex was significant on the levels of tryptophan (*P* = 0.002), and *UGT2B28* KO and male sex on the carbohydrate arabinose (*P* = 0.015).

The double KO individuals had distinct metabolic alterations within the same subclasses, with lower levels of several branched-chain amino acid and tryptophan metabolites such as indolelactate in males and kynurenine in females (Fig. 4D, Supplementary Table 2). Additionally, xylose was one of the most elevated metabolites (Fig. 4D). Females also displayed lower levels of pyrimidine nucleotides such as cytidine and of ascorbic acid (vitamin C) and its metabolites (Supplementary Table 2).

A substantial number of uncharacterized metabolites also exhibited distinct altered abundance in males and females of each KO group. This included 60% lower levels of X-19141, representing a highly discriminating metabolite in *UGT2B17* KO and double KO cases, irrespective of sex (*P*_*adj*_<0.05; Supplementary Tables 2, 4.2; Supplementary Fig. 4C). These metabolites were significantly influenced by the double KO status and sex, with males showing changes in X-21607 (*P* < 0.05) and females in X-21834 (*P* = 0.007).

### Metabolites impacted by UGT KOs are associated with common health conditions

**Fig. 5:**
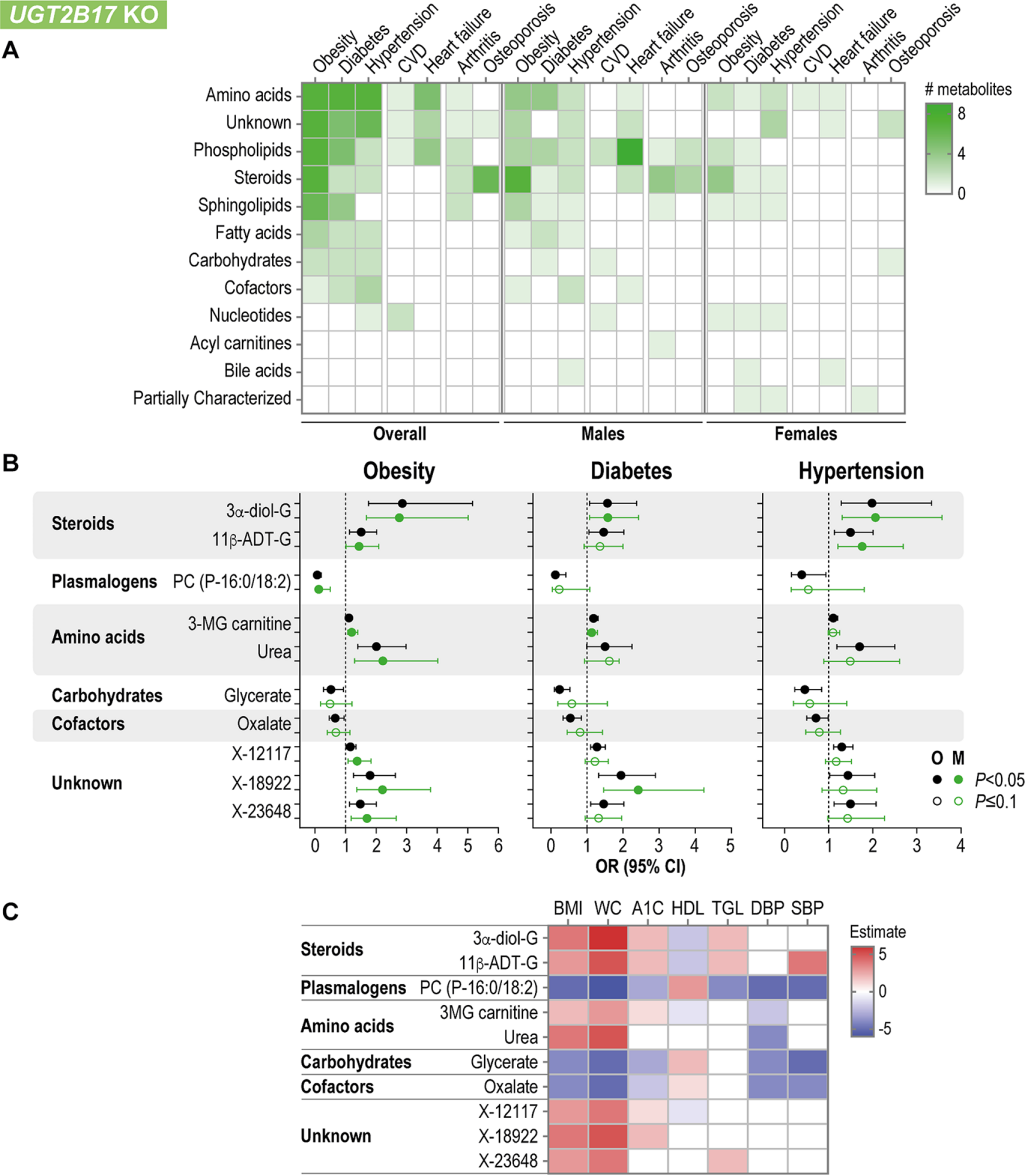
Association of *UGT2B17* KO divergent metabolites with the most common metabolic diseases and chronic conditions. **A**. Number of metabolites per subpathway significantly associated with diseases. CVD, cardiovascular diseases. **B**. Odds ratio (OR) and 95% confidence interval (CI) for the core metabolites commonly associated with obesity, hypertension and diabetes. 3α-diol-G, 5α-androstan-3α,17β-diol-17-glucuronide; 11β-ADT-G, 11β-hydroxyandrosterone glucuronide; PC (P-16:0/18:2), 1-(1-enyl-palmitoyl)-2-linoleoyl-glycerophosphocholine (P-16:0/18:2); 3MG carnitine, 3-methylglutarylcarnitine. Associations were significant (*P*_adj_<0.05) in a model adjusted for age, smoking and alcohol consumption. The overall analysis was also adjusted for sex. **C**. Significant associations (*P*_adj_<0.05) between the core metabolites and biomarkers related to obesity, diabetes and hypertension. BMI, body mass index; WC, waist circumference; A1C, hemoglobin A1c; HDL, high-density lipoprotein; TGL, triglycerides; DBP, diastolic blood pressure; SBP, systolic blood pressure

We examined the associations of significantly altered metabolites with prevalent metabolic diseases and chronic health conditions seen in the CLSA cohort (Supplementary Tables 1, 2). Obesity, diabetes and hypertension were associated with the largest numbers of altered metabolites (Fig. 5A). In particular, 10 metabolites belonging to a variety of metabolic classes were consistently associated with these conditions (Fig. 5B). They encompassed two androgen glucuronide conjugates, 3-methylglutaryl carnitine (a branched-chain catabolite of leucine), the monocarboxylic acid amide urea, and three unidentified metabolites, with elevated levels linked to increased disease risk. Conversely, elevated levels of the plasmalogen 1-(1-enyl-palmitoyl)-2-linoleoyl-glycerophosphocholine (P-16:0/18:2), glycerate, and the vitamin C derivative oxalate, were linked to a reduced risk of obesity, diabetes and hypertension (Fig. 5B). One of these core metabolites, namely the inactive androgen metabolite 5α-androstan-3α,17β-diol-17-glucuronide, is a direct product of the reaction catalyzed by UGT2B17 [43], whereas metabolites of other classes are not substrates of UGT2B17. Notably, certain metabolites were consistently associated with biomarkers of these diseases, such as BMI, waist circumference, blood pressure and blood levels of hemoglobin A1c, further lending strong support to these metabolite-disease associations (Fig. 5C).

A deeper examination of altered metabolites linked to the *UGT2B17* KO status revealed interconnectivity among other seemingly unrelated diseases (Supplementary Fig. 5). Higher levels of androgenic and pregnenolone steroids were associated with a lower risk of osteoporosis and arthritis, alongside obesity (Supplementary Fig. 5A, F, G). This observation is consistent with previous reports suggesting that UGT2B17 contributes to the pathogenesis of osteoporosis [21]. Similar to the elevated levels of sex steroids in *UGT2B17* KO, higher levels of phosphatidylcholine derivatives consistently correlated with a reduced risk of obesity, diabetes and heart failure (Supplementary Fig. 5A, B,E), whereas higher levels of sphingolipids were associated with an increased risk of obesity and arthritis (Supplementary Fig. 5A, G). These observations suggested a reduced risk of developing obesity for *UGT2B17* KO individuals, which aligns with studies reporting lower BMI for these individuals [44, 45]. Another example pertains to higher levels of glycine derivatives and lower tocopherol/vitamin E metabolites associated with a higher risk of obesity, diabetes and hypertension (Supplementary Fig. 5A–C).

Similar results were observed among female individuals lacking *UGT2B17*, with significant associations with obesity, hypertension and diabetes (Fig. 5A). In addition, metabolites that were elevated only in *UGT2B17* KO females were significantly associated with certain phenotypes; these included hexosylceramide glycosyl-N-tricosanoyl-sphingadienine (d18:2/23:0) linked to a reduced risk of obesity, diabetes and hypertension, the pyrimidine nucleotide N4-acetylcytidine linked to an increased risk of obesity, diabetes and hypertension, tetrahydrocortisol-glucuronide linked to a higher risk of obesity and diabetes, and higher levels of the uncharacterized metabolite X-12283 associated with a lower hypertension and increased risk of osteoporosis (Supplementary Fig. 6A-E). These relationships remained significant after adjustment for menopausal status and ever-use of HRT (Supplementary Fig. 6A-E).

**Fig. 6:**
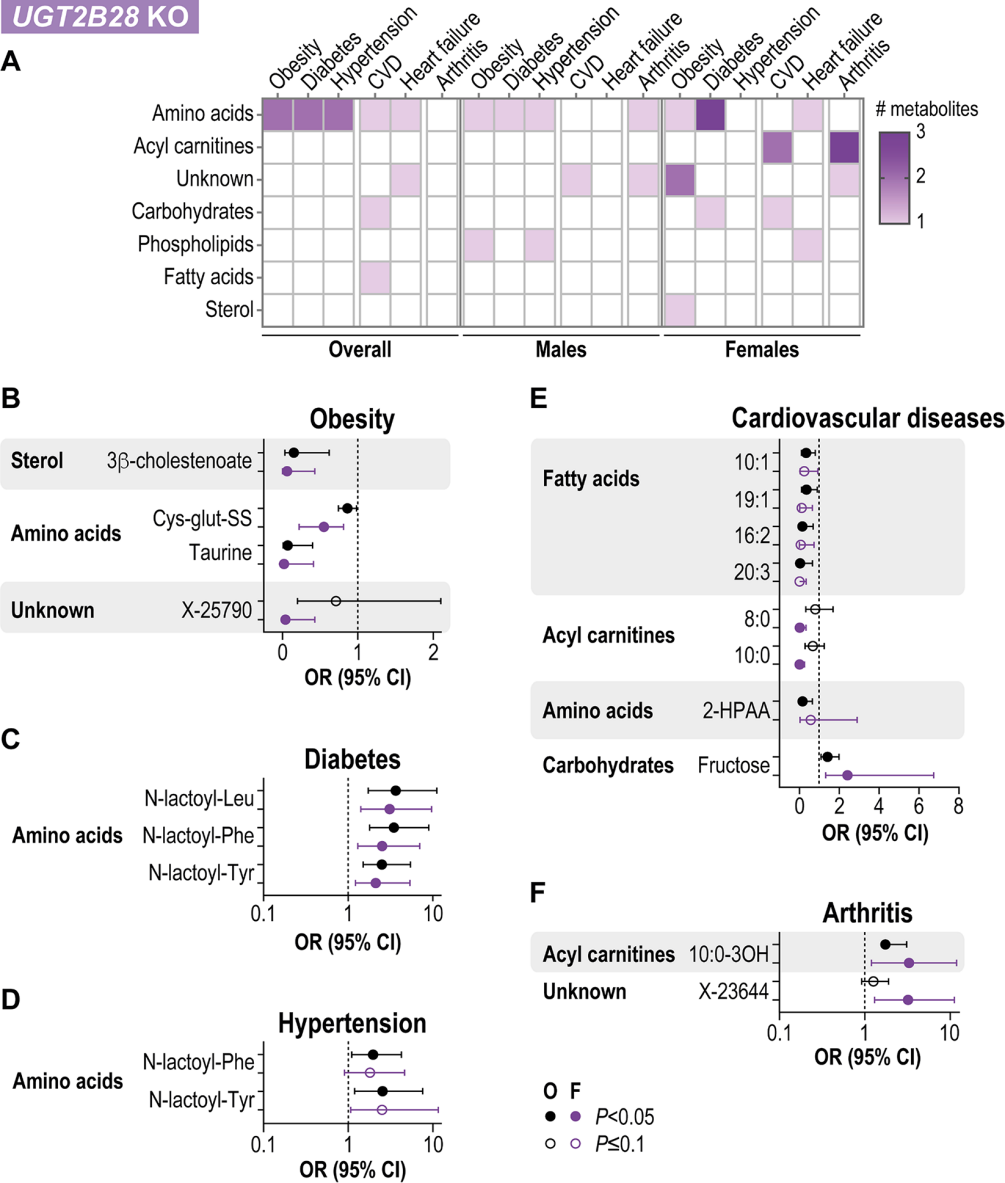
Metabolomic profile of *UGT2B28* KO is associated with chronic diseases. **A**. Number of metabolites per subpathway significantly associated with each disease. **B–F**. Odds ratio (OR) and 95% confidence interval (CI) for metabolites associated with obesity, hypertension, diabetes, cardiovascular diseases and arthritis. 3β-cholestenoate, 3β-hydroxy-5-cholestenoate; Cys-glutathione-SS, cysteine-glutathione disulfide; 2-HPAA, 2-hydroxyphenylacetate; N-lactoyl-Leu, N-lactoyl-leucine; N-lactoyl-Phe, N-lactoyl-phenylalanine; N-lactoyl-Tyr, N-lactoyl-tyrosine. Associations were significant (*P*_adj_<0.05) in a model adjusted for age, smoking and alcohol consumption, and for sex for the overall analysis. In *UGT2B28* KO females, after adjusting for these covariates as well as menopausal status and history of HRT use, all metabolites associated with obesity, diabetes, and X-23,644 in arthritis remained significant (*P*_adj_<0.05)

A comprehensive analysis of the altered metabolites in *UGT2B28* KO individuals revealed fewer significant associations compared with UGT2B17 deficiency (Fig. 6A). Metabolites belonging to the amino acid superpathway were particularly associated with obesity, diabetes, hypertension and cardiovascular diseases; these included higher levels of taurine associated with a reduced risk of obesity and BMI and of N-lactoyl derivatives of phenylalanine, leucine and tyrosine associated with an increased risk of diabetes and hypertension (Fig. 6B–D). These associations remained significant after adjustment for menopausal status and ever-use of HRT. For females, elevated levels of the glutathione derivative cysteine-glutathione disulfide, the sterol 3β-cholestenoate, and the unknown metabolite X-25790 were all linked to a reduced risk of obesity and inversely associated with BMI and waist circumference. After adjusting for menopausal status and ever use of HRT, most of the mentioned metabolites remained significant.

For *UGT2B28* KO females, several free fatty acids and the acyl carnitines octanoylcarnitine and decanoylcarnitine that were less abundant only in females were all linked to a reduced risk of cardiovascular diseases, whereas 3-hydroxy derivatives of decanoylcarnitine were associated with an increased risk of arthritis (Fig. 6E, F). Higher levels of fructose, which was one of the metabolites most enriched in plasma samples from *UGT2B28* KO females, were also associated with an increased risk of cardiovascular diseases (Fig. 6E). These relationships remained significant after accounting for menopausal status and ever-use of HRT. For *UGT2B28* KO males, characterized by globally lower levels of phospholipids, higher levels of 1-stearoyl-glycerophosphoethanolamine and 1-palmitoyl-2-stearoyl-glycerophosphatidylcholine were strongly linked to a lower risk of obesity and hypertension, whereas taurine, which was lower in males, was associated with a reduced risk of arthritis (Supplementary Fig. 6F).

## Discussion

Our results provide a comprehensive metabolomic profile of individuals with deletions of the metabolic UGT2B17 and UGT2B28 pathways, encoded by two of the most commonly deleted genes in the genome [3–5]. Despite the prevalence of these gene KOs in the population and the increasing number of diseases that have been linked to the complete deficiency of either enzyme in several small studies [21–24, 26, 27, 29, 46], research to date has predominantly focused on the association between *UGT2B17* and prostate cancer [19, 20, 24], with limited reports on *UGT2B28* [24]. Our study of the large CLSA cohort reveals the marked impact of each KO on many classes of metabolites, especially on specific lipid classes such as steroids and sphingolipids as well as amino acids, carbohydrates and uncharacterized metabolites, with important sex disparities. Systemic metabolic changes linked to each *UGT* KO were significantly associated, positively and negatively, with various health conditions including obesity, diabetes, hypertension, osteoporosis and arthritis, extending the potential impact of UGT deletions on health and disease. These findings underscore the potential importance of UGTs in maintaining metabolic balance and suggest their involvement in susceptibility to healthy aging and common health issues.

Our exploratory study included an unprecedentedly large number of individuals in each *UGT* KO group, enabling us to garner deep metabolic insights into the KO individuals. It also enabled an exploration of double KO, which revealed fewer significant alterations, likely due to its smaller sample size that limited the ability to detect changes. The age and sex distributions of the KO groups were consistent with those of the broader aging Canadian population, as reflected in the CLSA cohort [34]. One important hallmark of *UGT* KO individuals was their markedly altered blood lipid profiles and the clear unique impact on *UGT2B17* KO males and *UGT2B28* KO females. Probable contributors include the relatively high expression of *UGT2B17* in the liver of males [8, 9] and lower expression in gastrointestinal tissue [47] relative to females, alongside *UGT* expression in sex-specific organs (Supplementary Fig. 2). Steroid hormones, several of which serve as substrates for UGT2B17 and UGT2B28, may contribute to the observed sexual dimorphism [32, 33], a hypothesis supported by our data. For instance, *UGT2B17* KO males had reduced levels of androgen glucuronide conjugates that are known catalytic products of UGT2B17. A compensatory increase in androgen sulfate conjugates was also observed, as recently reported for *UGT2B17* KO male patients with prostate cancer [7]. The overall increased androgenic exposure observed in *UGT2B17* KO individuals underscores the pivotal role of the UGT2B17 pathway in regulating androgenic activity and androgen signaling. This does not imply that *UGT2B17* KO has no effect on the female steroid profile, as females had reduced levels of etiocholanolone-glucuronide. Profiling the blood metabolome—and more specifically two bile-acid glucuronides—also offers a method for monitoring individuals with *UGT2B17* KO, regardless of their sex. These metabolites, namely cholic acid and deoxycholic acid glucuronides, are end-products of the UGT2B17 enzymatic pathway [7] and were found to be linked to *UGT2B17* variants in recent genomic-metabolomic studies, as were two unknown metabolites in our study (X-24947 and X-19141) [48, 49]. For UGT2B28, despite substantial (>80%) amino acid sequence similarity with UGT2B17, the metabolomic profiles of deficient individuals were inversely impacted compared with those of *UGT2B17* KO, suggesting a distinct function for UGT2B28. UGT2B28 was initially reported to catalyze the conjugation of selected sex steroids but with much less efficiency compared with UGT2B17 [32], and UGT2B28 was found to be associated with a substantial accumulation of glucuronide derivatives in the CLSA cohort. This observation hints towards an inhibitory impact of UGT2B28 on glucuronidation that could be mediated by functional interaction with other UGTs, as observed for other UGT proteins [50, 51].

The two KOs we included in this study were defined using SNPs that are closely associated with deletion of *UGT2B28* or *UGT2B17.* SNP markers perfectly matched the *UGT2B28* deletion, whereas for *UGT2B17*, the genetic status relied on a SNP with strong but imperfect linkage disequilibrium (*R*^2^ < 0.8) [5]. Despite this limitation, some of our findings are supported by previous research reinforcing the validity of our findings. For example, the higher androgen abundance observed in *UGT2B17* KO individuals highlights the critical role of this pathway in regulating androgen bioavailability and androgen receptor signaling, especially in men, supported by its association with the progression of prostate cancer [52]. Thus, by associating UGT KOs with particular metabolic pathways and metabolites, the blood metabolome profiling provides molecular insights into the processes influencing disease pathogenesis, potentially linking UGT KOs to specific diseases. We further broadened our study to include associations with obesity, heart failure, osteoporosis and arthritis, in keeping with previous small size studies linking *UGT2B17* SNPs to body weight [44, 45] and as a susceptibility gene for osteoporosis [21]. Our findings for *UGT2B17* KO males suggest favorable metabolic health and possibly reduced prevalence of obesity, diabetes and hypertension. As for UGT2B28 deficiency, its impact on sex steroid levels was limited to progestins, which were elevated only in *UGT2B28* KO females. Although the association between progestins and osteoporosis, a female-predominant disease, did not reach significance for *UGT2B28* KO females, progestins were significantly associated vitamin D, a biochemical marker of osteoporosis, linked to a *UGT2B28* genetic variant in a recent genome-wide association study [53]. These observations support the physiological relevance of the metabolic changes caused by *UGT2B28* KO, particularly in females. The unexpectedly broad molecular divergence found among the *UGT* KO metabolomes implies their involvement in various pathways well beyond those specific to steroid hormones. It extends to metabolite subclasses that are not known UGT substrates, such as amino acids, fatty acids and carbohydrates. The mechanisms by which metabolites are affected remain to be demonstrated, potentially through direct enzymatic conjugation or other mechanisms that have yet to be characterized.

This study significantly broadened the range of metabolites impacted by UGT2B17 deficiency and identified several lipid subclasses (sphingolipids, phospholipids, carnitines) and amino acid derivatives that could be grouped into a core signature associated with metabolic diseases, namely hypertension, diabetes and obesity. Several of these metabolites —especially sphingolipids and phospholipids— were previously reported to be associated with risk of obesity, diabetes and hypertension [54–56]. In addition, recent genome-wide association studies identified *UGT2B17* as an effector gene for circulating levels of total cholesterol, apolipoprotein B [53], low density lipoprotein-cholesterol [52] and triglycerides [57] as well as associations between androgen levels and metabolic diseases [58]. In the case of UGT2B28-deficient individuals, the highly impacted fatty acids and amino-acid derivatives in women were closely associated with several metabolic disorders, an unexpected finding that opens a novel avenue of investigation regarding UGT2B28 function. For example, the positive association between elevated N-lactoyl amino acid blood levels and the risk of diabetes and hypertension is consistent with recent studies linking these amino acid derivatives to diabetic retinopathy and diabetes [42, 59]. Our findings suggest that both UGT2B17 and UGT2B28 are significant determinants of complex aging traits, while the potential compensatory roles of other genes warrant further investigations. Despite the unprecedented number of gene KO individuals included in the study, mostly Caucasians, it remains exploratory in nature, and further confirmatory research is required to establish broader clinical implications. A limitation of our study is the inability to comprehensively adjust for therapeutic interventions, which emphasizes the need for future studies to include detailed treatment information and investigate how therapies influence the effects of *UGT2B17* and *UGT2B28* deletions on the metabolome. Another limitation is the lack of diversity in age and menopausal status among the participants, as the majority were postmenopausal women. This may limit the applicability of our findings, particularly since the influence of *UGT2B17* and *UGT2B28* genetic status could differ in younger populations or premenopausal women.

## Perspectives and significance

This study reveals how natural and common germline *UGT* deletions —specifically *UGT2B17* and *UGT2B28*—disrupt metabolic balance and may contribute to sex-specific disease risks. These UGT enzymes are known to regulate steroids [32, 33]. By comparing individuals deficient in these UGTs to those who are proficient, we identified significant and distinct metabolic shifts associated with sex-specific disease susceptibilities, suggesting a broader role for these enzymatic pathways in shaping health outcomes. The variable frequencies of these gene deletions, which vary considerably based on ethnic backgrounds [3–5], underscore the need for further replication studies to validate our findings across diverse human populations, as our study was limited in terms of ethnic diversity. Existing literature supports a causal role, particularly for UGT2B17, in regulating androgen levels and influencing hormone-sensitive diseases such as prostate cancer progression [19, 20, 24]. Future studies investigating how UGT gene deletions interact with therapeutic interventions to influence the metabolome and disease outcomes could provide valuable insights for developing personalized medicine approaches. Our findings highlight the need for further studies to validate these associations, assess causal links, and explore potential applications in personalized medicine.

## Conclusion

By analyzing extensive population-based genomic and metabolomic data, our research emphasizes the crucial roles of UGT2B17 and UGT2B28 in shaping the systemic metabolome. Although these enzymes are primarily known for their role in steroid metabolism, our findings demonstrate that their influence extends to a wider array of metabolites, many of which are associated with both favorable and unfavorable effects on common health conditions and chronic diseases in a sex-divergent manner. This underscores a significant gap in our current understanding of their functions in both men and women across human populations, as well as the health implications related to common deficiencies in these genes.

## Electronic supplementary material

Below is the link to the electronic supplementary material.

Supplementary Material 1

Supplementary Material 2

## Data Availability

Data are available from the Canadian Longitudinal Study on Aging (www.clsa-elcv.ca) for researchers who meet the criteria for access to de-identified CLSA data.

## Declarations

### Disclaimer

The opinions expressed in this manuscript are those of the authors and do not reflect the views of the Canadian Longitudinal Study on Aging.

### Additional files

File name: Additional file 1

File format. Pdf.

Title of data: Supplementary Figures of Sexual Dimorphism in Metabolomic and Phenotypic Spectra of UGT deficiency: Findings from the Canadian Longitudinal Study on Aging.

Description of data:

### Supplementary Fig. 1

Metabolites quantified in this study and number of metabolites in each super-pathway that were available for analysis.

### Supplementary Fig. 2

Gene expression by sex for (**A**) *UGT2B17* and (**B**) *UGT2B28* in human tissues.

### Supplementary Fig. 3

Number of significantly altered metabolites in each UGT KO group relative to the gene-proficient reference group (*P* < 0.05) by subpathway.

### Supplementary Fig. 4

Relative abundance of significantly dysregulated metabolites in each KO group.

### Supplementary Fig. 5

Associations between significantly altered metabolites in *UGT2B17* KO and diseases.

### Supplementary Fig. 6

Associations between significantly altered metabolites in *UGT2B17* KO females, *UGT2B28* KO males, and diseases.

### File name: Additional file 2

File format. Excel.xlsx.

Title of data: Supplementary tables of Sexual Dimorphism in Metabolomic and Phenotypic Spectra of UGT deficiency: Findings from the Canadian Longitudinal Study on Aging.

Description of data:

### Supplementary Table 1

Detail of the chronic diseases / categories of chronic diseases used in this study.

### Supplementary Table 2

Fold change of the plasma metabolites measured in KO and reference groups.

### Supplementary Table 2.1

Significant changes in plasma metabolites for postmenopausal women.

### Supplementary Table 3

Characteristics of the study groups.

### Supplementary Table 4

Top 5 most changed metabolites in UGT2B17 KO, UGT2B28 KO and Double KO for overall and sex-based analyses.

### Supplementary Table 4.1

Top 5 most changed metabolites including unknown and partially characterized metabolites in UGT2B17 KO, UGT2B28 KO and Double KO for overall and sex-based analyses.
