# Supplementary material for: Sexual dimorphism in metabolomic and phenotypic spectra of UGT deficiency: findings from the Canadian Longitudinal Study on Aging
Source: Biol Sex Differ. 2025 Apr 22;16:26. doi: 10.1186/s13293-025-00708-5 (PMC12013048; doi:10.1186/s13293-025-00708-5)
Supplement: Supplementary file 1 — Supplementary Material 1 [file 13293_2025_708_MOESM1_ESM.pdf]

Sexual Dimorphism in Metabolomic and Phenotypic Spectra of UGT deficiency: Findings from the Canadian Longitudinal Study on Aging

Ana Lucia Rivera-Herrera[a,b,c], Michèle Rouleau[a,b,c], Mahukpe Narcisse Ulrich Singbo[c], Tania Cuppens[b,c,d], Julien Prunier[b,c,d], Arnaud Droit[b,c,d], David Simonyan[c], Chantal Guillemette[a,b,c,e†]

[a]Faculty of Pharmacy, Université Laval, Québec, QC, Canada, G1V 0A6

[b]Université Laval Cancer Research Center, Québec, QC, Canada, G1R 3S3

[c]Centre Hospitalier Universitaire (CHU) de Québec Research Center - Université Laval, Québec, QC, Canada, G1V 4G2

[d]Faculty of Medicine, Department of Molecular Medicine, Université Laval, Québec, QC, Canada, G1V 0A6

[e]Canada Research Chair in Pharmacogenomics, Université Laval

Supplementary Figures

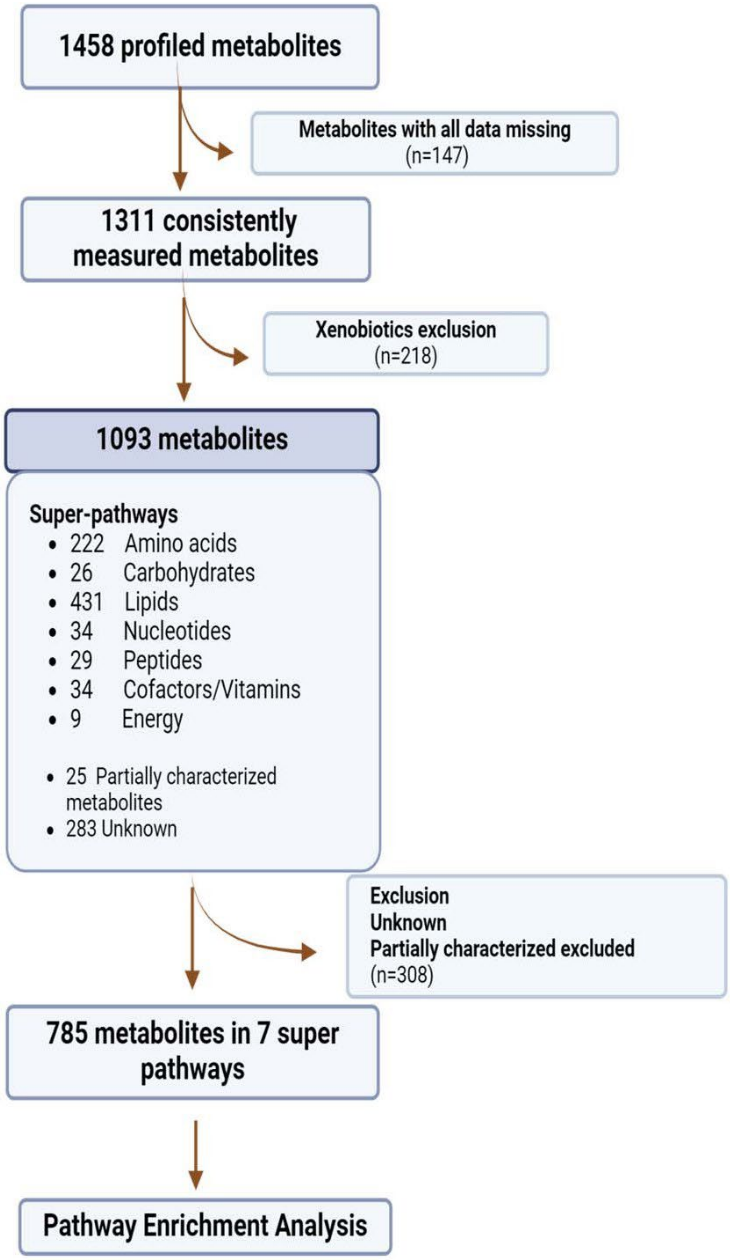

Supplementary Figure 1. Metabolites quantified in this study and number of metabolites in each super-pathway that were available for analysis. Created with Biorender.com.

A UGT2B17

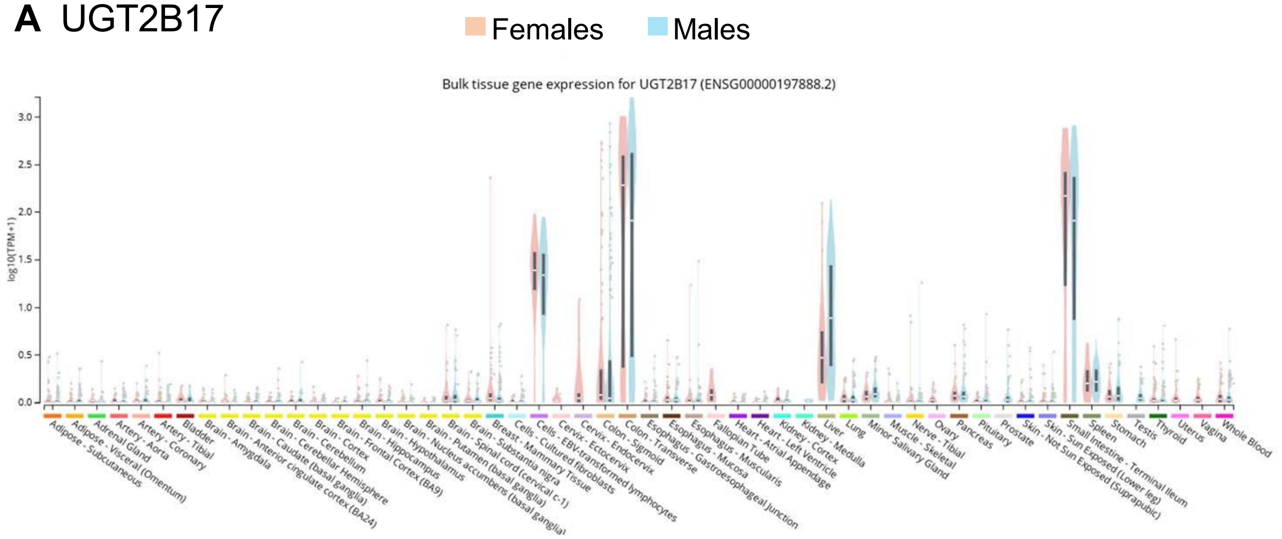

B UGT2B28

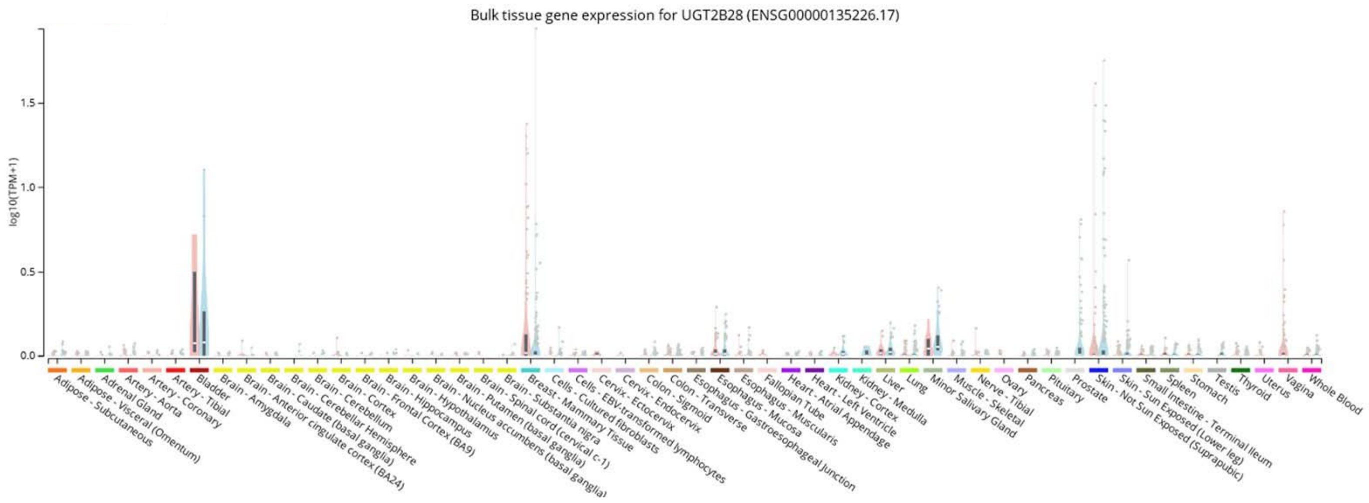

Supplementary Figure 2. Gene expression by sex for (**A**) *UGT2B17* and (**B**) *UGT2B28* in human tissues. Data are from GTEx Analysis Release V8 (dbGaP Accession phs000424.v8.p2), accessed on 2024/01/15.

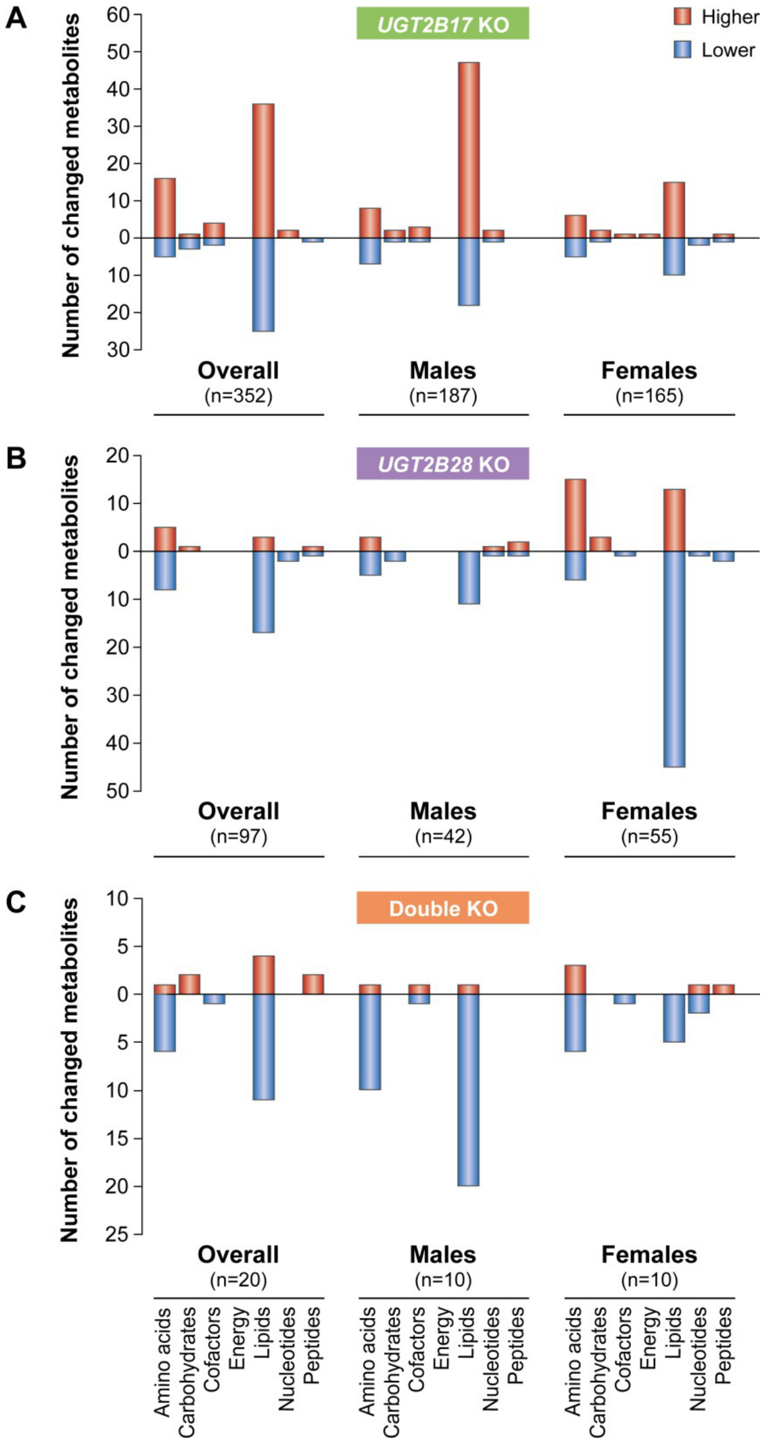

Supplementary Figure 3. Number of significantly altered metabolites in each UGT KO group relative to the gene-proficient reference group (P<0.05) by sub-pathway.

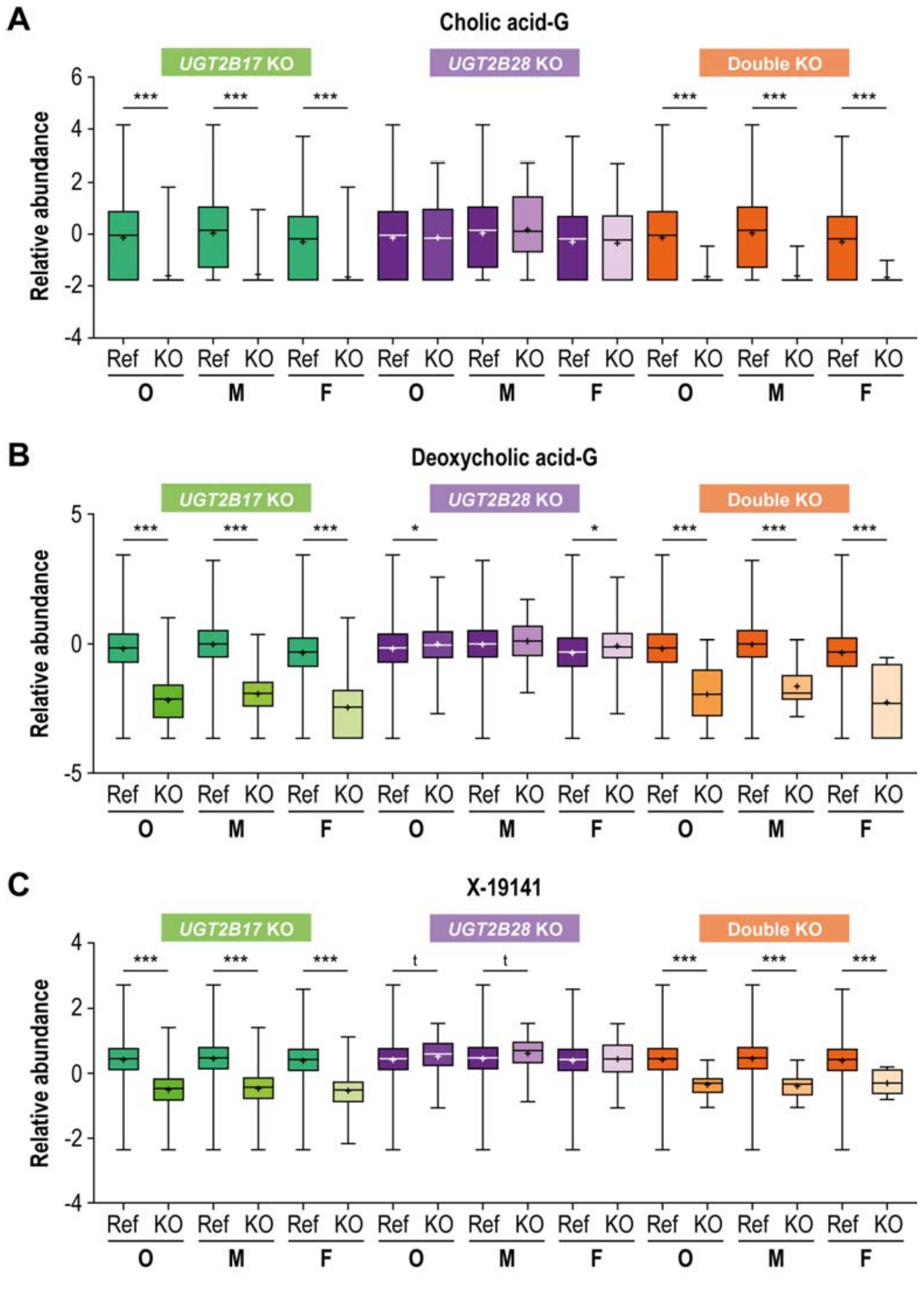

Supplementary Figure 4. Relative abundance of significantly dysregulated metabolites in each KO group. Abundance of (**A**) cholic acid glucuronide (-G), (**B**) deoxycholic acid-G, and (**C**) X-19141 was significantly lower in all *UGT2B17* KO individuals. Boxes represent interquartile range, the median (horizontal bar), and mean (+). Whiskers depict minimum and maximum values. Ref, reference group. **P*<0.05, ****P*<0.001, t, *P*≤0.1. Overall (O), male (M) and female (F) analyses are shown.

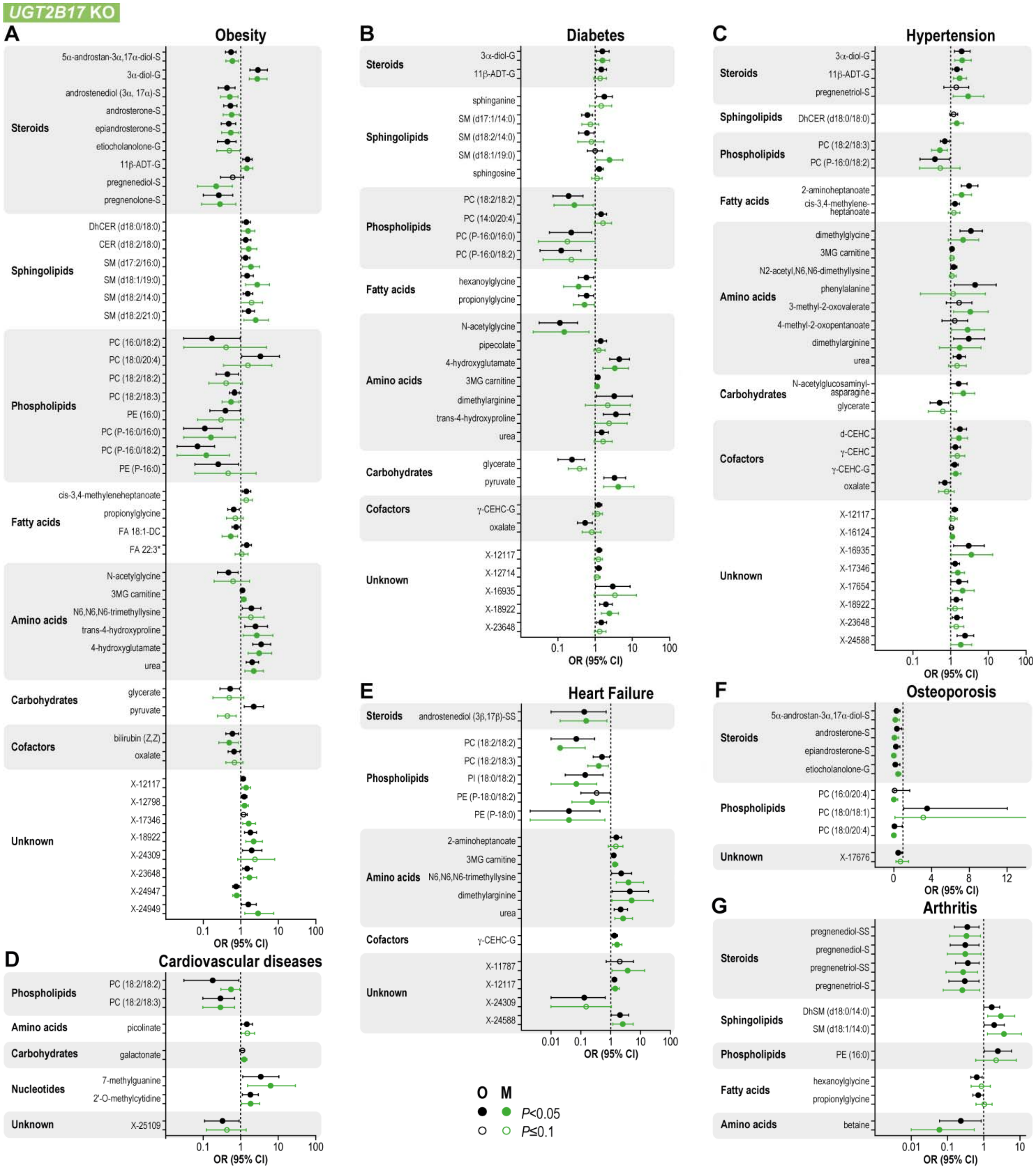

Supplementary Figure 5. Associations between significantly altered metabolites in *UGT2B17* KO and diseases. Odds ratio (OR) and 95% confidence interval (CI) are shown for significant associations with (**A**) obesity, (**B**) diabetes, (**C**) hypertension, (**D**) cardiovascular diseases, (**E**) heart failure, (**F**) osteoporosis, and (**G**) arthritis for *UGT2B17* KO overall (black) and males (green). Associations were significant (P_{adj}<0.05) in a model adjusted for age, smoking, and alcohol. The overall analysis was also adjusted for sex. 3α-diol-G, 5α-androstan-3α,17β-diol 17-glucuronide; 11β-ADT-G, 11β-hydroxyandrosterone glucuronide; 3MG carnitine, 3-methylglutarylcarnitine; -S, monosulfated; -SS, disulfated; -G, glucuronide; DhSM, dihydrosphingomyelin; DCER, dihydroceramide; CER, ceramide; SM, sphingomyelin; PC, phosphatidylcholine; PE, phosphatidylethanolamine; PI, phosphatidylinositol; FA, fatty acids. The 22:3* and 22:5* denote omega 6 polyunsaturated fatty acids. FA 18:1-DC, octadecenedioate.

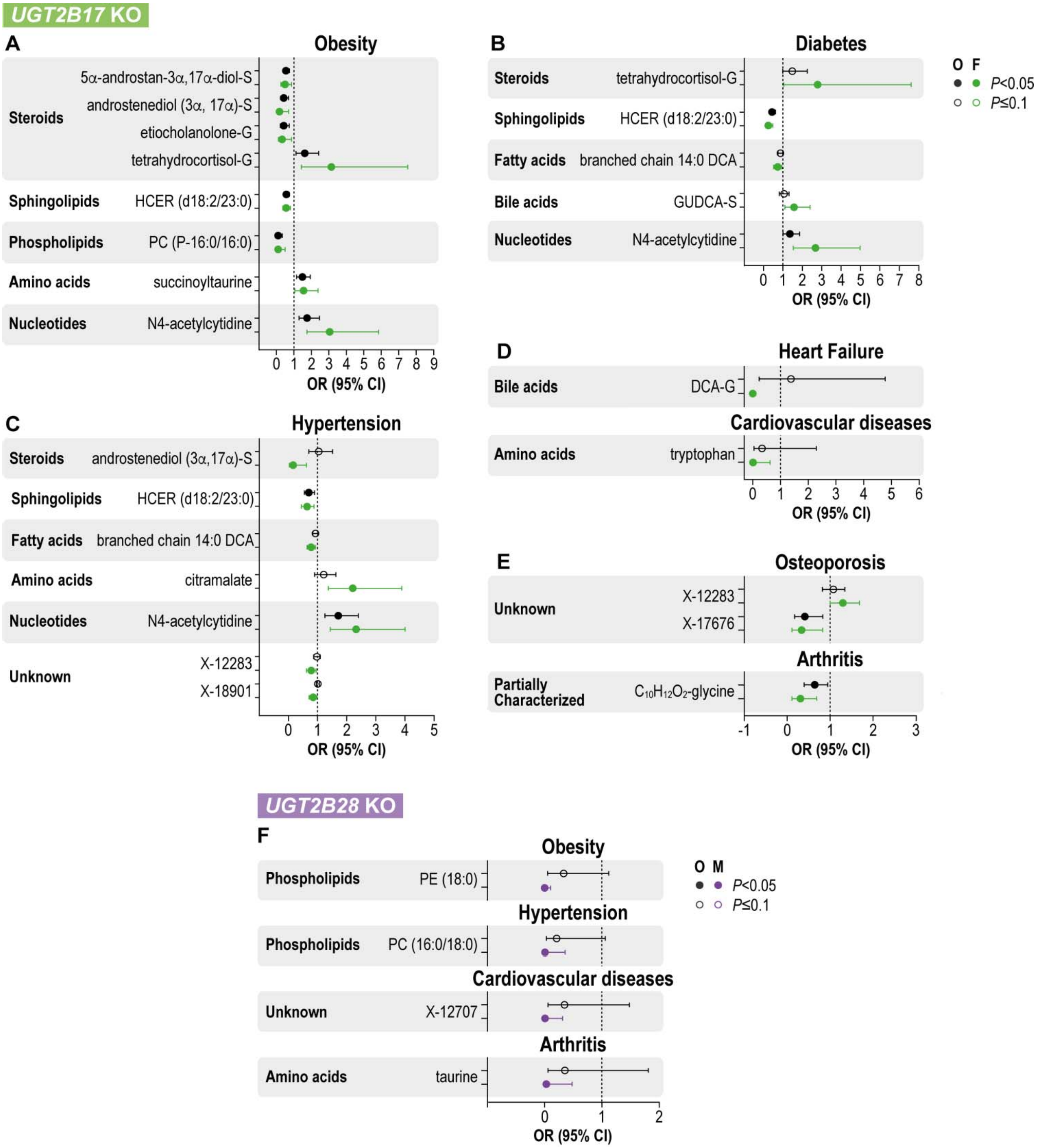

Supplementary Figure 6. Associations between significantly altered metabolites in *UGT2B17* KO females, *UGT2B28* KO males, and diseases. Odds ratio (OR) and 95% confidence interval (CI) are shown for significant associations of *UGT2B17* KO females with (**A**) obesity, (**B**) diabetes, (**C**) hypertension, (**D**) heart failure and cardiovascular diseases, and (**E**) osteoporosis and arthritis, and for *UGT2B28* KO males with (**F**) obesity, hypertension, cardiovascular diseases, and arthritis. Associations were significant (P_{adj}<0.05) in a model adjusted for age, smoking, and alcohol. In the model adjusted for menopausal status and ever use of HRT as covariates in addition to for age, smoking, and alcohol, all metabolites showed significant associations (P_{adj}<0.05) except for DCA-G in heart failure and X-12283 in osteoporosis. Branched-chain 14:0 DCA, branched-chain 14:0 dicarboxylic acid; -S, monosulfated; -SS, disulfated; -G, glucuronide; HCER, hexosylceramides; PC, phosphatidylcholine; PE, phosphatidylethanolamine; GUDCA-S, glycoursodeoxycholic acid sulfate; DCA-G, deoxycholic acid glucuronide.
